# Predicting the effects of parasite co-infection across species boundaries

**DOI:** 10.1098/rspb.2017.2610

**Published:** 2018-03-14

**Authors:** Joanne Lello, Susan J. McClure, Kerri Tyrrell, Mark E. Viney

**Affiliations:** 1 School of Biosciences, Cardiff University, Cardiff CF10 3AX, UK; 2 Department of Biodiversity and Molecular Ecology, Research and Innovation Centre, Fondazione Edmund Mach, S. Michele all'Adige, Trentino 38010, Italy; 3 Division of Animal, Food and Health Sciences, CSIRO, Armidale, New South Wales 2350, Australia; 4 School of Biological Sciences, University of Bristol, Bristol BS8 1TQ, UK

**Keywords:** co-infection, population dynamics, immune response, immunomodulation, nematode, helminths

## Abstract

It is normal for hosts to be co-infected by parasites. Interactions among co-infecting species can have profound consequences, including changing parasite transmission dynamics, altering disease severity and confounding attempts at parasite control. Despite the importance of co-infection, there is currently no way to predict how different parasite species may interact with one another, nor the consequences of those interactions. Here, we demonstrate a method that enables such prediction by identifying two nematode parasite groups based on taxonomy and characteristics of the parasitological niche. From an understanding of the interactions between the two defined groups in one host system (wild rabbits), we predict how two different nematode species, from the same defined groups, will interact in co-infections in a different host system (sheep), and then we test this experimentally. We show that, as predicted, in co-infections, the blood-feeding nematode *Haemonchus contortus* suppresses aspects of the sheep immune response, thereby facilitating the establishment and/or survival of the nematode *Trichostrongylus colubriformis*; and that the *T. colubriformis*-induced immune response negatively affects *H. contortus*. This work is, to our knowledge, the first to use empirical data from one host system to successfully predict the specific outcome of a different co-infection in a second host species. The study therefore takes the first step in defining a practical framework for predicting interspecific parasite interactions in other animal systems.

## 1. Introduction

Co-infecting parasite species can interact with one another, potentially altering both within-host infection dynamics [1–3] and between-host transmission (e.g. by increasing or decreasing parasite reproductive output or by altering host susceptibility) [2,4–7]. In turn, changes in infection dynamics within hosts can alter host disease severity and/or duration [8–10] and may directly or indirectly confound attempts to control parasite infection [3,11,12]. In most cases, whether or not particular parasite species interact, and the nature of such interactions are unknown. Despite the important consequences of co-infection, the potential interactions among parasites are, therefore, rarely considered in either clinical settings or during the design of infection control programmes. One possible solution to this problem would be to discover and define rules that determine when and how parasites interact. Such a concept has been explored at a broad scale for macroparasite–microparasite interactions using a meta-analysis of different infection combinations in mice [13]. This meta-analysis demonstrated that macroparasite–microparasite co-infection would normally result in increased numbers of microparasites owing to helminth-induced impairment of the anti-microparasite immune response, but that such effects would be moderated where resource competition was also present. This was a seminal contribution to the field of co-infection biology, highlighting the potential to predict co-infection using easily obtained parasite traits. However, because of the necessarily broad categorizations in this analysis, and the focus on a single model host system, application of these findings in a clinical or public health setting is difficult. Two key questions therefore follow logically from this meta-analysis: (i) can predictions also be made at a species-specific scale appropriate for use in clinical and public health settings? and (ii) can patterns of parasite interspecific interaction be robustly predicted across different host species?

In earlier work, we demonstrated, using previously published data, that if parasites were grouped according to both the immune responses they stimulate and those which affect them [14], it was possible to predict the result of a co-infection. This approach was limited, however, by the necessity for detailed immunological data for each of the co-infecting parasites. Here, we develop and extend this approach by using taxonomic and parasite niche traits (i.e. resource use, site of infection) to assign parasite species to groups, making the assumption that organisms assigned to these groups will interact with the host immune system in a similar manner to one another. Subsequently, we infer what the immune interaction of each parasite group will be with its host, and hence the likely immune relationship between the groups, based on a known example of a co-infection interaction between representative species from those groups.

In a previous study of the parasite community of wild rabbits (*Oryctolagus cuniculus*), we described a range of interspecific interactions, including the interaction between two gut nematodes; the blood-feeding stomach worm *Graphidium strigosum* and the intestinal worm *Trichostrongylus retortaeformis*, a mucosal browser [3]. We showed that an increasing abundance of *G. strigosum* was associated with increased infection intensity of *T. retortaeformis* but, conversely, that the presence of *T. retortaeformis* was associated with a reduced intensity of *G. strigosum*. We further proposed that these effects occurred because (i) *G. strigosum* downregulated anti-worm immune response in the host, and *T. retortaeformis* was given an advantage by this suppression, while (ii) *T. retortaeformis* induced an immune response which, though reduced in co-infection, acted against *G. strigosum* [3]. In sheep, there are parasite species that are taxonomically and functionally equivalent to the parasite groups found in the rabbit; specifically, the nematode *Haemonchus contortus*, which lives in the abomasum (stomach) of the sheep and feeds on host blood, and *Trichostrongylus colubriformis*, which lives downstream in the small intestine and feeds on the host mucosa. We predict that these two parasites of sheep will interact with the same pattern, and by the same process, as the functionally equivalent parasite species in the rabbit. This is, to our knowledge, the first empirical attempt to predict the consequences of a hitherto untested interspecific interaction and to do so using data from different host and parasite species.

Not all parasitic nematodes are equal in the immune responses that they stimulate, or that affect them [15,16]. While the immune control of the majority of gut nematodes is associated with a T-helper cell 2 (Th2) immune response [17,18], many nematodes are able to subvert this response to varying degrees. Such immunomodulation may be particularly important for blood-feeding species. These nematodes are usually very harmful to their host, causing both tissue damage and anaemia, with heavy infections sometimes proving fatal [19]. In addition, blood-feeding nematodes are frequently found at a high prevalence in their host populations [20,21]. Therefore, it would be reasonable to expect hosts to evolve strong immune responses against blood-feeding nematodes. Yet age-prevalence and age-intensity curves for these parasites show that they cause chronic infections and/or repeatedly reinfect the host [20], suggesting that immune responses are functionally unsuccessful against them. Furthermore, many blood-feeding nematode species have been shown to have wide-ranging immunomodulatory capacities (e.g. *Ancylostoma duodenale*, *Ancylostoma caninum, Necator americanus*, *Angiostrongylus cantonensis, H. contortus* [22–26]). While these species do induce a strong Th2 response [23,27], many simultaneously subvert that response through a range of mechanisms [28]. These immunomodulatory effects may have consequences for other co-infecting parasite species. In contrast to blood-feeding nematode species, *Trichostrongylus* spp. browses on intestinal mucosa and bacteria, and shows limited invasion and penetration into host tissues [29]. These nematodes tend to produce shorter-lived infections than those of blood-feeding species, being more rapidly and effectively controlled by the host [30,31]. While there is evidence that *Trichostrongylus* spp. may have some immunomodulatory capacity, it does not appear to be as immunologically broad ranging as that observed among the blood-feeding species [16,32]. Further evidence of the different immune responses to these parasite groups is seen in rabbits, where the temporal pattern of natural and laboratory infections suggests that *T. retortaeformis* is effectively removed by the host, while *G. strigosum* is not [3,33]. In summary, we therefore propose that how these two parasite groups interact with their hosts' immune responses will result in predictable interspecific interactions.

Here, we test our hypothesis in sheep experimentally co-infected with *H. contortus* and *T. colubriformis* (comparing them to sheep mono-infected with each species, and with uninfected controls), by measuring nematode intensity and the host immune response. We specifically predict that in co-infections (i) the blood-feeding *H. contortus* will suppress aspects of the host immune response, thereby facilitating the establishment and/or survival of *T. colubriformis*, and (ii) the *T.* colubriformis-induced immune response will negatively affect *H. contortus*.

## 2. Material and methods

### (a) Pre-infection protocol

**Figure 1.**
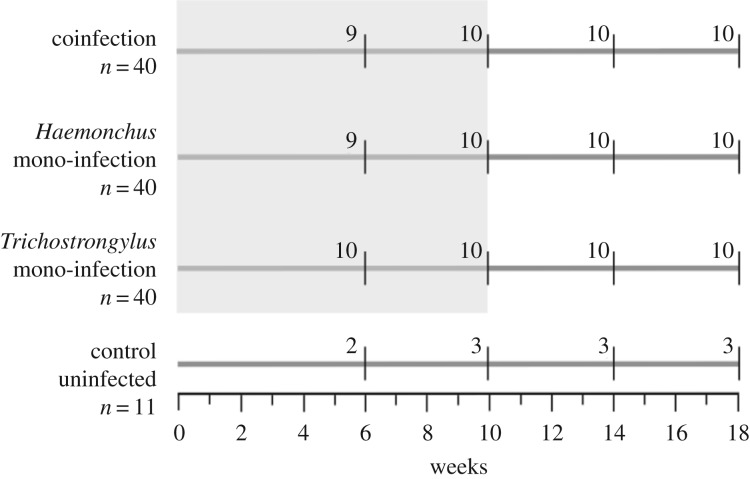
A schematic of the experimental protocol. Co-infection and mono-infection groups of animals were infected twice weekly for 10 weeks (shaded box) and the animals were then sampled (10 per infection group, and three for the control group) after 6, 10, 14 and 18 weeks post-initial infection.

Following approval by the FD McMaster Laboratory, Chiswick, Animal Ethics Committee, at weaning, 132 Merino wethers (castrated rams) were brought into CSIRO Livestock Industries animal house where faecal samples were analysed using a modified McMaster technique (as in [34]) to diagnose any helminth infection. Animals were then treated with a mixture of Abamectin and Praziquantel, Levamisole and Benzimidazole, using the manufacturers’ recommended doses. Twelve days later a second faecal screen for helminth infection was performed to confirm that animals were helminth-free. All animals were blood-sampled via jugular venepuncture to provide a pre-infection baseline immune and health status measure. Animals were then assigned to one of four treatment groups using a stratified random assignment (where groups were balanced for body mass, body condition and original faecal egg count). The four treatment groups were: (i) control, uninfected (*n* = 12), (ii) *H. contortus* mono-infected (*n* = 40), (iii) *T. colubriformis* mono-infected (*n* = 40), or (iv) *H. contortus* and *T. colubriformis* co-infected (*n* = 40) (figure 1).

### (b) Infections and sampling

An overview of the experimental protocol is shown in figure 1. Animals in the co-infected and mono-infected groups were each infected twice weekly for 10 weeks with 300 larvae of *H. contortus* and/or 1500 larvae of *T. colubriformis*. For animals in the co-infection groups, doses of both parasite species were given simultaneously as an additive dose. Differential dosing was used because of the different size and pathogenicity of the two helminth species, *T. colubriformis* being considerably smaller and less pathogenic than *H. contortus* [35]. Animals in the control, uninfected group, were handled in the same manner as other animals. Throughout the experiment animals were maintained on raised slatted floors to prevent self-reinfection, provided fresh water ad libitum, and fed daily with a ration of 700 g of standard pellets consisting of lucerne (500 g kg^−1^), wheat (100 g kg^−1^), pollard (200 g kg^−1^), bran (160 g kg^−1^), salt (20 g kg^−1^) and ammonium chloride (20 g kg^−1^), the quantity of which was set for normal growth.

At weeks 6, 10, 14 and 18 post-initial infection (where initial infection indicates the first day of larval dosing), all animals were blood-sampled, as above, and body mass and body condition (assessed using the industry-standard scale of 0–5, www.lifetimewool.com.au/conditionscore.aspx) were recorded. At each of these four sample points, a subset of animals (10 for each infection group, and three for the control, uninfected group) was humanely slaughtered and tissue collected, and processed as described below.

### (c) Worm counts

From killed animals, the abomasum and small intestine were sampled in sections, placed into separate dissecting trays, the tissue opened and the contents gently washed into collecting jars to remove all adult nematodes. The number of worms in subsamples was then counted to determine the total number of worms of each species infecting each animal. Samples of abomasal and jejunal tissues (4 cm^2^ squares) were fixed in Bouin's solution for later histological analysis. *Haemonchus contortus* larvae can developmentally arrest within the host at the L4 stage, a form of diapause known as hypobiosis. Hypobiosis does not occur in the strain of *T. colubriformis* used in our study. Remaining abomasal tissue was, therefore, digested in phosphate-buffered saline containing 10% v/v HCl to release any arrested *H. contortus* fourth-stage larvae, which were then counted.

### (d) Measures of immune response

We measured the number of immune cells in the fixed abomasal and jejunal tissue, which, following standard sectioning, were stained with haematoxylin and eosin, and toluidine blue [36]. For both tissue samples, cell counts and scores were estimated per villus–crypt unit (i.e. from the tip of one villus to the next). For the abomasal tissue, we determined the number of globule leucocytes, mast cells and eosinophils, and scores for lymphocyte infiltration (0 = no infiltration, to 4 = heavy infiltration). For jejunal tissue the same cell counts and scores were made, but in addition the number of goblet cells and a score of the proportion of goblet cells containing granules (0 = no cells contained granules, to 5 = most cells contained granules) were also recorded, together with a score of the thickness of the smooth muscle layer (0 = very thin, to 4 = thick).

We determined the concentration of IgG1 antibodies against *H. contortus* and against *T. colubriformis* L3 antigens using previously described enzyme-linked immunosorbent assays (ELISAs) [36,37].

### (e) Statistical analyses

One animal was removed from the control group prior to infection because of ill health, leaving a control group sample size of 11 animals. One animal was also removed from each of the co-infection and *H. contortus* mono-infection groups prior to the 6-week sample point, due to ill health unrelated to the helminth infections, leaving a sample size of 39 sheep for each of these two groups. A small number of other sampling losses owing to processing problems are detailed in the electronic supplementary material, S1, which provides an overview of sample size by sample point for all analyses.

Analyses were conducted in R v. 3.1.2 [38]. The effect of infection treatment group on the number of adult *T. colubriformis* worms, the number of adult *H. contortus* worms and the number of *H. contortus* arrested larvae were assessed in three general linear models (GLMs). Infection group (mono- or co-infected), days post-initial infection (i.e. cull day; included as a categorical variable) and their interaction were included as independent variables. In addition, the faecal egg count pre-anthelminthic treatment and animals' total gain in mass were also accounted for by inclusion as independent terms. Following preliminary model assessments, the number of arrested larvae of *H. contortus* was square root transformed (sqrt(*x* + 1)) to normalize the residuals of that GLM. Neither Poisson nor negative binomial error distributions provided better model fits for any model (electronic supplementary material, S2).

We used two steps to determine how treatment group affected the measures of immune responses in the abomasum and jejunum. First, two principal components (PCs) analyses were conducted separately on the abomasal and jejunal measures of immune responses, using a singular value decomposition of the centred and scaled data matrix [39]. All scores were treated as numeric data and scaling was applied. The measures of the abomasal immune responses were compared between the *H. contortus* mono- and co-infection groups; and measures of the jejunal immune responses were compared between *T. colubriformis* mono- and co-infection groups; in both cases, this separation reflects the location of these species within the animals. The PC explaining the majority of the variation in each analysis was then used as the dependent variable in a GLM where treatment group, time of sampling and their interaction were the explanatory variables. Models were refined in a stepwise manner by evaluating the *F* statistics (terms were rejected when *p* > 0.05). Where the GLM analyses showed significant differences in PC values between treatment groups, the second step in the analysis was undertaken. In these second analyses, the bootstrapped mean value was calculated for each individual measure of immune response, to qualitatively explore the effect of treatment group on these individual measures. For the treatment groups, bootstrapped mean values were calculated for each time of sampling. For the uninfected control animals, the data were pooled across sample points due to the smaller sample size in this group.

The effect of treatment group on anti-*H. contortus* and anti-*T. colubriformis* IgG1 titres were assessed in two general linear mixed models (GLMMs using the R package ASReml-R v3.0) in which each animal's individual identification number was included as a random term to control for pseudo-replication. The titres of IgG1 were transformed to normalize residuals in the model, as ((*x* + 1)^0.12^) for anti-*T. colubriformis* and ((*x* + 1)^0.18^) for anti-*H. contortus* responses. Results shown here are back-transformed. In these models, treatment group, time of sampling (included as a categorical variable) and their interaction were included as fixed effects. This fixed-effect model was refined in a stepwise manner using the Wald test and evaluation of the conditional *F* statistics (terms were rejected when *p* > 0.05). Where treatment group was found to be a significant effect, differences between treatment groups were assessed by within-model contrasts.

## 3. Results

### (a) Co-infection affects *Trichostrongylus colubriformis* and *Haemonchus contortus*

**Figure 2.**
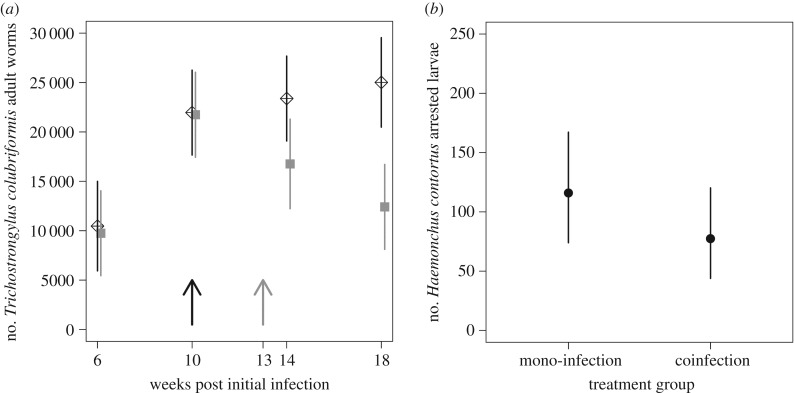
Effect of co-infection on within-host parasite dynamics. The predicted number of (*a*) *T. colubriformis* adult worms by time post-initial infection and infection group and (*b*) *H. contortus* hypobiosed larvae by infection group. Error bars are the 95% confidence intervals. In (*a*), the *T. colubriformis* mono-infection group is denoted by the closed grey squares, and the co-infection group by the crossed diamonds; the black arrow represents the last day of larval dosing and the grey arrow represents the first day by which the last larval dose may potentially have reached adulthood. Groups have been offset by one day to aid visualization.

*Trichostrongylus colubriformis* was a more successful parasite of sheep when it was in a co-infection with *H. contortus* (the number of adult *T. colubriformis* differed between the co-infection and mono-infection groups through time post-initial infection *F*_3,69_ = 3.38, *p* = 0.023; figure 2). There were more adult *T. colubriformis* worms in co-infected sheep than in *T. colubriformis*-only infections at 14 and 18 weeks post-initial infection (*t*_69_ = −2.08, *p* = 0.041; *t*_69_ = −3.96, *p* < 0.001, respectively). A total of 30 000 *T. colubriformis* infective larvae were given to each sheep, which by week 14 could all have developed into adult worms. In the co-infected animals a mean of 23 380 adults were present (78%), whereas only 16 761 (56%) were found in the *T. colubriformis*-only infections (see the electronic supplementary material, S3 for mean and s.d. of raw counts through time).

*Haemonchus contortus* was also affected by co-infection, but differently compared with *T. colubriformis*. To assess the *H. contortus* infection, we analysed both the number of arrested L4-stage larvae in the host tissues along with adult worms (see the electronic supplementary material, S3 for mean and s.d. of raw counts through time). There were fewer *H. contortus* arrested larvae in co-infections, compared with *H. contortus*-only infections (*F*_1,71_ = 4.15, *p* = 0.045; figure 2); the number of these larvae was also affected by the time post-initial infection (*F*_3,71_ = 9.79, *p* < 0.001; electronic supplementary material, S4). By contrast, the number of adult *H. contortus* was not affected by co-infection, though numbers did vary through time post-initial infection (*F*_3,72_ = 14.73, *p* < 0.001; electronic supplementary material, S5). As the number of adults show no evidence of being bolstered by larvae leaving the arrested state in the co-infection group, together these data mean that in co-infections there are overall fewer *H. contortus* worms.

### (b) Co-infection affects host cellular immune responses

**Figure 3.**
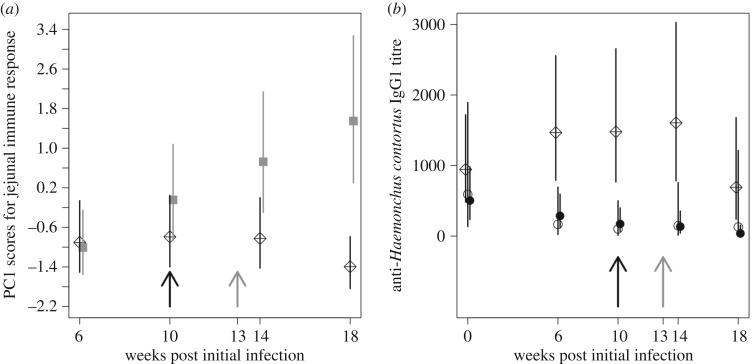
Immune responses during co-infection. (*a*) The predicted PC1 scores of jejunal immune response, with time post-initial infection and *T. colubriformis* infection group (i.e. mono- and co-infection). The *T. colubriformis* mono-infection group is denoted by the closed grey squares and the co-infection group is denoted by the crossed diamonds. (*b*) Predicted anti-*H. contortus* IgG1 titre concentration through time post-initial infection for the control (open black circles), *H. contortus* mono-infection (solid black circles) and co-infected (crossed diamonds) groups. In (*a,b*), groups have been offset by one day to aid visualization. Error bars are the 95% confidence intervals. The black arrow represents the last day of larval dosing and the grey arrow represents the first day by which the larvae from the last dose may have reached adulthood.

**Figure 4.**
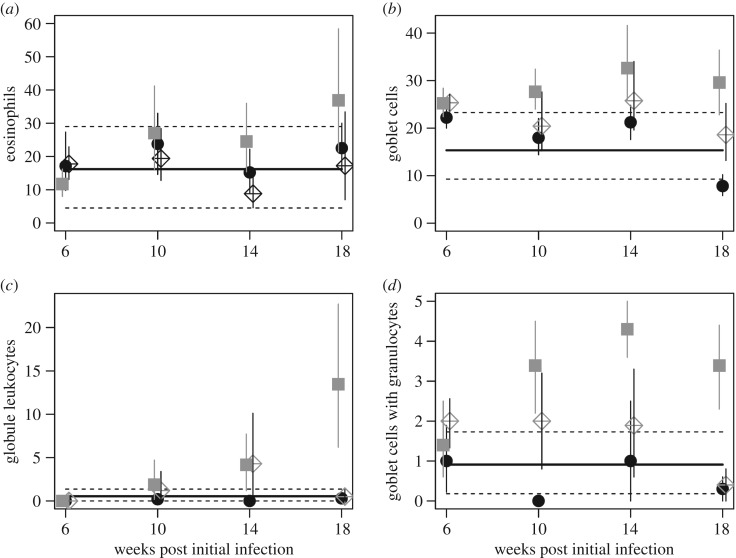
Jejunal immune responses are shown as the bootstrapped number (per villus–crypt unit) of (*a*) eosinophils, (*b*) goblet cells, (*c*) globule leucocytes and (*d*) score of goblet cells with granules. Treatment groups have been offset by 1 day to aid visualization. Error bars are the 95% confidence intervals. The solid black line and dashed lines represent the bootstrapped mean for the control treatment group and its 95% confidence intervals, respectively. Grey squares represent *T. colubriformis* mono-infection, solid black circles *H. contortus* mono-infection and crossed diamonds co-infection.

*Trichostrongylus colubriformis* infects the jejunum and to measure the immune responses in this site, we used a PC analysis of jejunal immune measures. All immune measures positively loaded onto PC axis 1 (PC1), which explained 49% of the variance in these components (electronic supplementary material, S6). PC1 was subsequently used in the GLM analysis and transformed (Ln(PC1 + 3)), resulting in a normal distribution of the model residuals; the results shown in the figures are back-transformed. The PC1 scores significantly differed between the co-infection and mono-infection groups, through time post-initial infection (GLM analysis of PC1 scores *F*_3,71_ = 3.84, *p* = 0.013; figure 3). The PC1 scores for the co-infected group did not vary with time post-initial infection, whereas those of the mono-infected group increased through time. The predicted PC1 values in the co-infected animals were significantly lower than in the *T. colubriformis*-only infection group (significant difference between co-infected and mono-infected group at weeks 14 and 18 post-initial infection *t*_71_ = 2.32, *p* = 0.023, *t*_71_ = 4.50, *p* < 0.001). Together, this means that the jejunal immune response induced by *T. colubriformis* was suppressed in co-infected animals. Analysis of the individual cell types in the jejunum also showed that the greatest responses were in the *T. colubriformis*-only infection group and lower in the co-infected animals, presumably owing to the immunosuppressive effect of *H. contortus* (figure 4; electronic supplementary material, S7). In animals mono-infected with *H. contortus*, the jejunal immune responses were often as low as those in the control (uninfected) animals, which is unsurprising given that *H. contortus* is not present in the jejunum. *Haemonchus contortus* infects the abomasum, and to measure the immune responses in this site we used a PC analysis of the abomasel immune measures. All abomasel immune measures loaded positively onto PC1 explaining 62% of the variance (electronic supplementary material, S8). PC1 was subsequently used in the GLM analysis and transformed (Ln(PC1 + 3)), resulting in a normal distribution of the model residuals; the results shown in the figures are back-transformed. GLM analyses of the abomasal PC1 scores showed that they did not differ significantly between the co-infected and mono-infected animals, nor did they vary through time post-initial infection.

### (c) Co-infection increases anti-*Haemonchus contortus* larval immune responses

The concentration of anti-*H. contortus* IgG1 was significantly different between co-infected and *H. contortus*-only infected animals (effect of treatment group excluding the *T. colubriformis* mono-infection group *F*_8,300_ = 3.31, *p* = 0.001; figure 3). The response was significantly greater in the co-infected animals, compared with the *H. contortus*-only infected and control animals, which did not differ from one another (figure 3). In the co-infected animals at 18 weeks post-initial infection the IgG1 response was reduced, coinciding with a reduced number of arrested *H. contortus* larvae (electronic supplementary material, S4).

The concentration of anti-*T. colubriformis* IgG1 was significantly affected by treatment group (effect of treatment group, excluding the *H. contortus* mono-infection group, *F*_8,300_ = 3.09, *p* = 0.002; electronic supplementary material, S9). Specifically, these responses were significantly higher in the co-infected and *T. colubriformis*-only infection groups compared with the control, uninfected group. The co-infected and *T. colubriformis*-only infection groups were not significantly different from one another (electronic supplementary material, S9).

## 4. Discussion

We hypothesized that, by defining parasite groups using taxonomy and parasite traits, we could infer the host response to those groups and hence the expected interaction among co-infecting parasites. Our hypothesis was supported. Specifically, we demonstrate that immune suppression by the blood feeder *H. contortus* had a positive effect upon the numbers of mucosal browser *T. colubriformis*, while the immune response promoted by the mucosal browser negatively affected the numbers of the blood feeder.

## 5. Effect of the blood feeder on the mucosal browser

The presence of *H. contortus* resulted in comparatively more *T. colubriformis* adult worms in co-infected sheep. The trajectory of adult worm numbers in the *T. colubriformis* mono-infected sheep shows a classic convex age-intensity curve, indicative of host immune responses removing adult worms [30,40,41]. In the co-infection treatment group the number of worms reached an asymptote, suggesting that adult worms were not being removed by the host immune response. There was, however, some evidence of a reduction in the larval establishment in this co-infection group (though less than in the mono-infected group), probably indicating that an anti-*T. colubriformis* response was beginning to develop. This is consistent with previous studies that have shown the anti-*T. colubriformis* immune response acts first against incoming larvae [42].

As we hypothesized, the difference in the number of *T. colubriformis* adults between co-infection and mono-infection groups appears to be immune-mediated. Our data demonstrate that there was a reduced immune response in the jejunum in the co-infected animals, compared to the *T. colubriformis* mono-infected animals, and most pronounced in the latter time points (weeks 14 and 18 post-initial infection; figures 3 and 4 and electronic supplementary material, S7). This differentiation between the infection groups suggests that the immune suppression we observe is dependent on the adult *H. contortus* (since by week 14 all larvae would have developed to adulthood or arrested their development). We use the presented immune measures as general indicators of anti-helminth immune responses, rather than implicating individual immune components. Nevertheless, all these immune components have been associated with the immune response against helminths in sheep [43–45].

## 6. Effect of the mucosal browser on the blood feeder

There was no evidence of an effect of co-infection on the number of *H. contortus* adults, nor on the abomasal cellular immune response. However, the significantly fewer arrested larvae in the co-infected animals demonstrate that co-infection still has a negative effect on *H. contortus* (figure 2). In natural infections, arrested larvae resume development to adulthood during periods of host stress [36]. There are significantly less arrested larvae in the co-infection group but no more adults. These missing larvae must, therefore: (i) be lost to the system entirely, or (ii) have replaced adults that have been lost. Thus, these larvae either (a) never established in the arrested state in the first place, (b) were destroyed in, or expelled from, the tissues, or (c) following a period in the arrested state, resumed their development and either replaced lost adults, or failed to establish as adults. The difference in the number of larvae found in the arrested state between singly and co-infected groups of sheep is relatively small, approximately 40 larvae, and is thus unlikely to be of clinical significance in these sheep. We highlight, however, that this study is not focused upon clinical significance per se, but upon the ability of our predictive framework to establish the form and direction of the parasite interactions, which we have achieved. Nevertheless, even these few larvae, as adults, could contribute substantially to the potential infectious burden on pasture under natural conditions. Assuming an average daily fecundity of 4700 eggs per female [46] and a sex ratio of 1 : 1, 20 adult female worms could be adding more than 94 000 eggs per day to pasture.

As predicted, the loss of *H. contortus* arrested larvae appears to be immune-mediated. Although the abomasal immune components do not differ among infection groups, the concentration of anti-*H. contortus* IgG1 was significantly higher in co-infected animals (figure 3). *Haemonchus contortus* larvae were the source antigen for the IgG1 assay and it is likely that this antibody response reduces larval development, as has been previously been reported [47].

## 7. Is the observed interaction robust?

The host immune response to *T. colubriformis* and *H. contortus* in mono-infections is well documented [48–51]. A feature of these responses is that they differ in strength depending on host species (sheep or goat), breed [17], [52–54], age [30], [55] and diet [56], although the same immune components are implicated in helminth control among these host groups. An important consideration, then, is whether the interactions we have described between the co-infecting parasite groups would be robust to such host differences. As the immune components involved in the host response are the same, we suggest that while there may be quantitative differences in intensity of infection owing to variation in the strength of the immune response, the qualitative result (i.e. positive consequences for a mucosal-browsing nematode and negative for the blood-feeding group) will probably persist. This view is further supported by the identical pattern of interaction seen in the rabbit co-infection system between its blood-feeding and mucosal-browsing nematode parasites. It should be noted that one laboratory study of co-infection with the same rabbit helminths did not find this pattern of interaction during co-infection [33]. That laboratory study, however, used a single, high-dose infection (rather than the trickle infections we used), which can dramatically alter the form of the elicited immune response [57], in turn altering the nature of the interspecific interactions.

Our hypothesis for the interaction between the sheep nematodes was based on data from a different host and different parasite species, where we defined parasite groups based on their taxonomic and parasitological (i.e. resource use, site of infection) traits. We suggest that this novel approach can be more generally applied to other host and parasite systems. While we have successfully applied this approach here, we acknowledge that this is a single test and that further work is required to confirm that the approach could be applied beyond our defined parasite groupings. However, we note that our predictive ability crossed host species (rabbits and sheep) that are distinct taxonomically, behaviourally and physiologically, suggesting that host similarity does not underlie our successful prediction. Regarding the parasites, we also emphasize that our hypothesis of how the sheep parasites would interact came solely from our predictive framework. Specifically, despite extensive prior study of these parasites in sheep, the interactions we correctly predicted had never previously been hypothesized. Together this suggests that our predictive framework is neither host nor parasite species-specific. Future exploration of this topic could include a meta-analysis to determine whether parasite traits can represent patterns of immune function across multiple host types and different forms of parasite (i.e. beyond helminths).

Notably, the parasite species in our study all belong to the superfamily Trichostrongyloidea and it is possible that the interaction observed would be restricted to species within this superfamily – though this would still be an important result. Nevertheless, we have described here the common immunomodulatory features of several blood-feeding nematode species, which further supports this parasite grouping and also proposes a mechanism (i.e. suppression of the intestinal cellular immune response) for this groups' potential interaction with other parasite groups. There is less information available to support the grouping of mucosal-browsing nematodes, as the host immunological response to this group has been less well studied. Even if we narrow this group to mucosal-browsing *Trichostrongylus* spp., the only immune function studies conducted appear to be on *T. colubriformis* and *T. retortaeformis*, the species involved in our studies. It will therefore be interesting to determine whether other members of the group also stimulate, and are controlled by, a classic Th2 response, which underlies the mechanism of their interaction with the blood feeders and, further, whether the group could be expanded to other helminth species displaying similarly low levels of tissue invasion, i.e. browsing nematodes beyond *Trichostrongylus* spp.

We propose that the form of acquisition of a given resource is likely to be an important indicator of how the host will respond to any parasite. For example, while nematodes and malaria both use the host blood as a resource, they acquire that resource in a different way. We suggest that taxonomically more related parasites are also more likely to evolve related mechanisms of resource acquisition and therefore that a combined grouping strategy involving location, resource use and parasite taxonomy may be a good indicator of host immune response, the ultimate mechanism of the interspecific parasite interaction in our study. Our classification mechanism requires that the resource use of the parasite is known. For some species, this will not be the case. However, using physical location in conjunction with taxonomic similarity to other known species will often be a suitable proxy.

## 8. Implications for parasite control and economic losses

*Haemonchus contortus* and *T. colubriformis* are both economically important parasites, causing substantial production losses in both sheep and goats [58]. Production losses owing to *T. colubriformis* are likely to be greater in sheep co-infected with *H. contortus*, because of the higher worm burdens and prolonged infection in such co-infections. Notably, the condition and mass of co-infected animals did not significantly differ from the other treatment groups. However, pasture-reared sheep, not provisioned with the high-quality maintenance diet provided in our experiment, would probably experience more severe effects during co-infection. Transmission of *T. colubriformis* in co-infected sheep could be substantially higher owing to the higher worm burdens and prolonged infection during *H. contortus* co-infection, meaning potentially higher worm burdens at a population level, requiring the use of anthelmintics. However, density-dependent reduction in *per capita* worm fecundity has been observed for *T. colubriformis* [59], which may ameliorate such effects. Nevertheless, host immune response appears to play a role in this density-dependent restriction of fecundity [17], and thus such immune effects may be reduced during *H. contortus* co-infection. A change in *H. contortus-*induced production losses during co-infection are unlikely, as adult worm burdens of this species were not affected by the co-infection. The economic implications of this co-infection are, therefore, principally a consequence of the altered dynamics of the *T. colubriformis* infection.

## 9. Conclusion

This work represents, to our knowledge, a first experimental proof-of-principle that groups of parasites can be identified and thereafter used to predict the outcome of a previously unexplored interspecific parasite interaction in a different host species. Given the ubiquity and multiplicity of co-infection in nature, it is important that we derive such grouping mechanisms. In previous work, we suggested grouping parasites by an immunological profile [14]. A problem with this idea is that immune profiling is complex and expensive, and reagents may not be available for a novel or lesser-studied hosts. However, the current study offers an alternative mechanism for classification by using taxonomy and more easily identified parasitological traits, to act as a proxy for the immune traits. Further, we have demonstrated that we can successfully use these traits to predict the immunologically based interaction of two parasite groups. This work therefore proposes a general framework for predicting the relationships between other parasite groups, and next steps should be to determine how widely applicable such a framework can be.

## Supplementary Material

S1 Table

## Supplementary Material

S2 Table

## Supplementary Material

S3 Table

## Supplementary Material

S4 Figure

## Supplementary Material

S5 Figure

## Supplementary Material

S6 Table

## Supplementary Material

S7 Figure

## Supplementary Material

S8 Table

## Supplementary Material

S9 Figure

## Data accessibility

All the data described in this study are available from the Dryad Digital Repository: https://doi.org/10.5061/dryad.1802hj0 [60].
