# Supplementary material for: Predicting the effects of parasite co-infection across species boundaries
Source: Proc Biol Sci. 2018 Mar 14;285(1874):20172610. doi: 10.1098/rspb.2017.2610 (PMC5879626; doi:10.1098/rspb.2017.2610)
Supplement: S1 Table [file rspb20172610supp1.docx]

**S1 Number of samples processed per treatment group for each type of analysis.** a) Adult worm and arrested larval counts. b) Cellular immune response analyses. c) IgG1 titre analyses, which was a repeated measures analysis involving a pre-infection baseline bleed (time 0) hence sample size reduces as animals were removed for sampling.

| a) Fig 2 main text, table S2 and S3 and figures S4 & S5 | | | | |
| --- | --- | --- | --- | --- |
|  | Treatment Groups | | | |
| Time point | Control | *T. colubriformis* mono-infection | *H. contortus* mono-infection | Coinfection |
| 42 | NA | 10 | 9^#^ | 9^#^ |
| 70 | NA | 10 | 10 | 10 |
| 98 | NA | 9**^§^** | 10 | 10 |
| 126 | NA | 10 | 9**^§^** | 9**^§^** |
| b) Fig 3a & Fig 4 main text, PCA Tables S6 and S8 and figure S7 | | | | |
|  | Treatment Groups | | | |
| Time point | Control | *T. colubriformis* mono-infection | *H. contortus* mono-infection | Coinfection |
| 42 | 2^#^ | 10 | 9^#^ | 9^#^ |
| 70 | 3 | 10 | 10 | 10 |
| 98 | 3 | 10 | 10 | 10 |
| 126 | 3 | 10 | 10 | 10 |
| c) Fig 3b main text, and figure S6 | | | | |
|  | Treatment Groups | | | |
| Time point | Control | *T. colubriformis* mono-infection | *H. contortus* mono-infection | Coinfection |
| 0 | 11^#^ | 40 | 39^#^ | 39^#^ |
| 42 | 11 | 40 | 39 | 39 |
| 70 | 9 | 30 | 30 | 30 |
| 98 | 6 | 19^\|\|^ | 20 | 20 |
| 126 | 2^\|\|^ | 9^\|\|^ | 10 | 10 |

^Reduced sample due to # animal being removed from experiment due to ill health, unrelated to the experimental infections,^ **^§^** ^worm data lost due to breakage during transport, || failed ELISA assay^
