# Supplementary material for: Predicting the effects of parasite co-infection across species boundaries
Source: Proc Biol Sci. 2018 Mar 14;285(1874):20172610. doi: 10.1098/rspb.2017.2610 (PMC5879626; doi:10.1098/rspb.2017.2610)
Supplement: S2 Table [file rspb20172610supp2.docx]

**S2 Generalised Linear Model residual distributions.** Shapiro tests for normality of model residuals for each generalised linear model starting model of worm and larval count data are presented comparing the Gaussian error distribution with identity link, the Poisson error distribution with Log link and the negative binomial distribution with log link. The comparison shows that in each case the model using the Gaussian distribution provides a superior fit with normally distributed errors. Model formula = Dependent variable ~ treatment group + time post initial infection + starting FEC + weight gain + treatment group : time post initial infection (where : represents an interaction between terms).

| **Dependent variable** | **Error Distribution**  **(link function)** | **W statistic** | **P value** |
| --- | --- | --- | --- |
| *Trichostrongylus colubriformis* adult parasite count | Gaussian  (identity) | 0.975 | 0.093 |
|  | Poisson  (log) | 0.904 | <0.001 |
|  | Negative binomial  (log) | 0.904 | <0.001 |
| *Haemonchus contortus* adult parasite count | Gaussian  (identity) | 0.980 | 0.263 |
|  | Poisson  (log) | 0.888 | <0.001 |
|  | Negative binomial  (log) | 0.895 | <0.001 |
| *Haemonchus contortus* arrested larval count | Gaussian  (identity)  Response variable sqrt(x+1) transformed | 0.993 | 0.963 |
|  | Poisson  (log) | 0.922 | <0.001 |
|  | Negative binomial  (log) | 0.915 | <0.001 |
