# Supplementary material for: Predicting the effects of parasite co-infection across species boundaries
Source: Proc Biol Sci. 2018 Mar 14;285(1874):20172610. doi: 10.1098/rspb.2017.2610 (PMC5879626; doi:10.1098/rspb.2017.2610)
Supplement: S3 Table [file rspb20172610supp3.docx]

**S3 Mean helminth counts by week post initial infection.** Standard deviations (SD) are shown in brackets. The boostrapped mean (resampling 10000 times with replacement) is shown in italicised text for *Trichostronglus colubriformis* single infection at week 18 post initial infectionas the data at this time point were overdispersed.

|  | *Trichostrongylus colubriformis* adults (SD) | | *Haemonchus contortus* adults (SD) | | *Haemonchus contortus* arrested larvae (SD) | |
| --- | --- | --- | --- | --- | --- | --- |
| Weeks post initial infections | Single infection | Coinfection | Single infection | Coinfection | Single infection | Coinfection |
| 6 | 9745  (2372) | 10467 (2631) | 639  (553)* | 719  (364) | 106  (73) | 114  (82) |
| 10 | 21740 (3861) | 21955 (5647) | 1580  (452) | 1568  (836) | 125  (44) | 100  (85) |
| 14 | 16761 (6366) | 23380 (5647) | 1750  (713) | 2090  (698) | 225  (141) | 125  (131) |
| 18 | 12350  *16582*  (14029) | 25011 (8023) | 1733  (643) | 2308  (892) | 44  (51) | 25  (25) |
