# Supplementary figures and images for: Predicting the effects of parasite co-infection across species boundaries
Source: Proc Biol Sci. 2018 Mar 14;285(1874):20172610. doi: 10.1098/rspb.2017.2610 (PMC5879626; doi:10.1098/rspb.2017.2610)

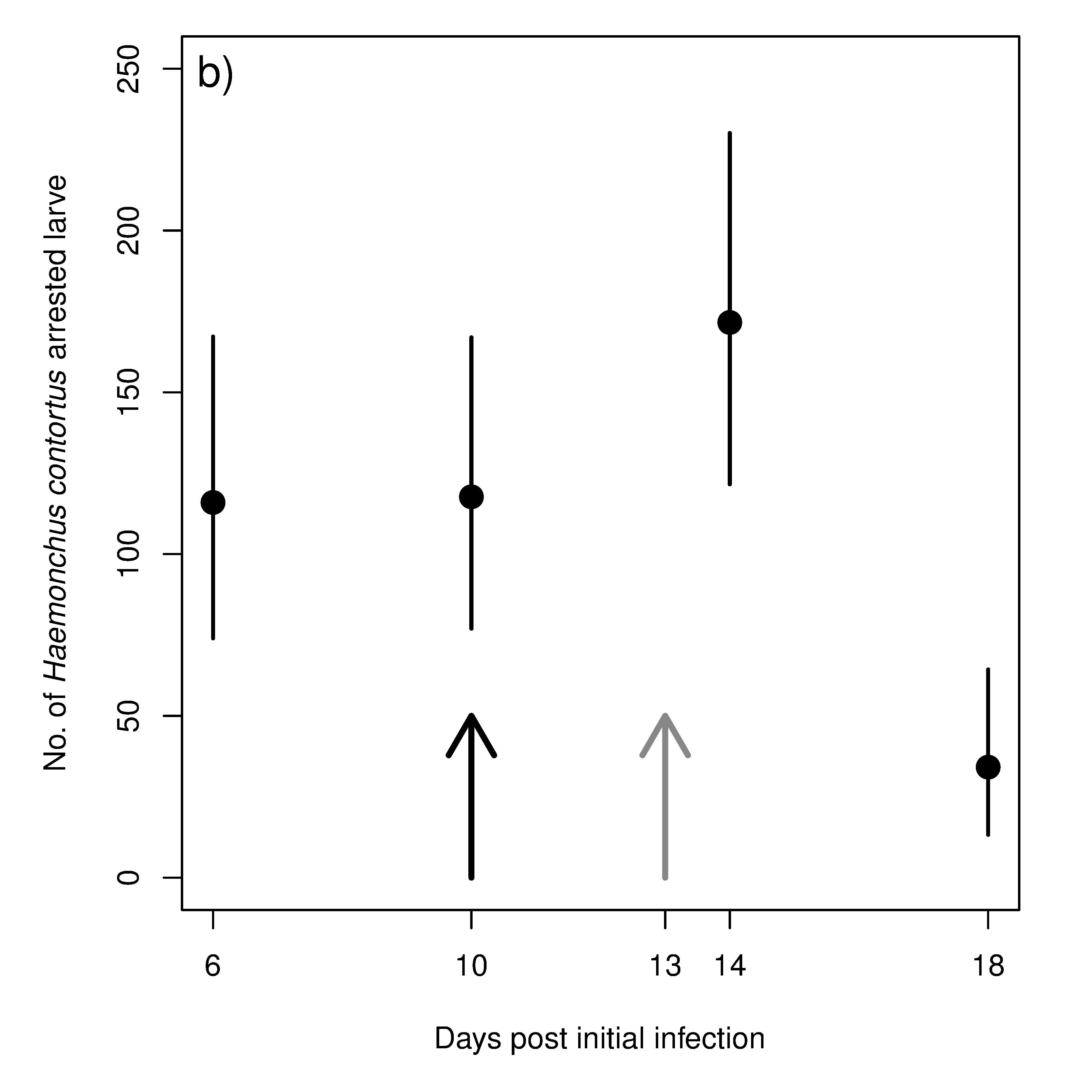

Supplement: S4 Figure [file rspb20172610supp4.tif]

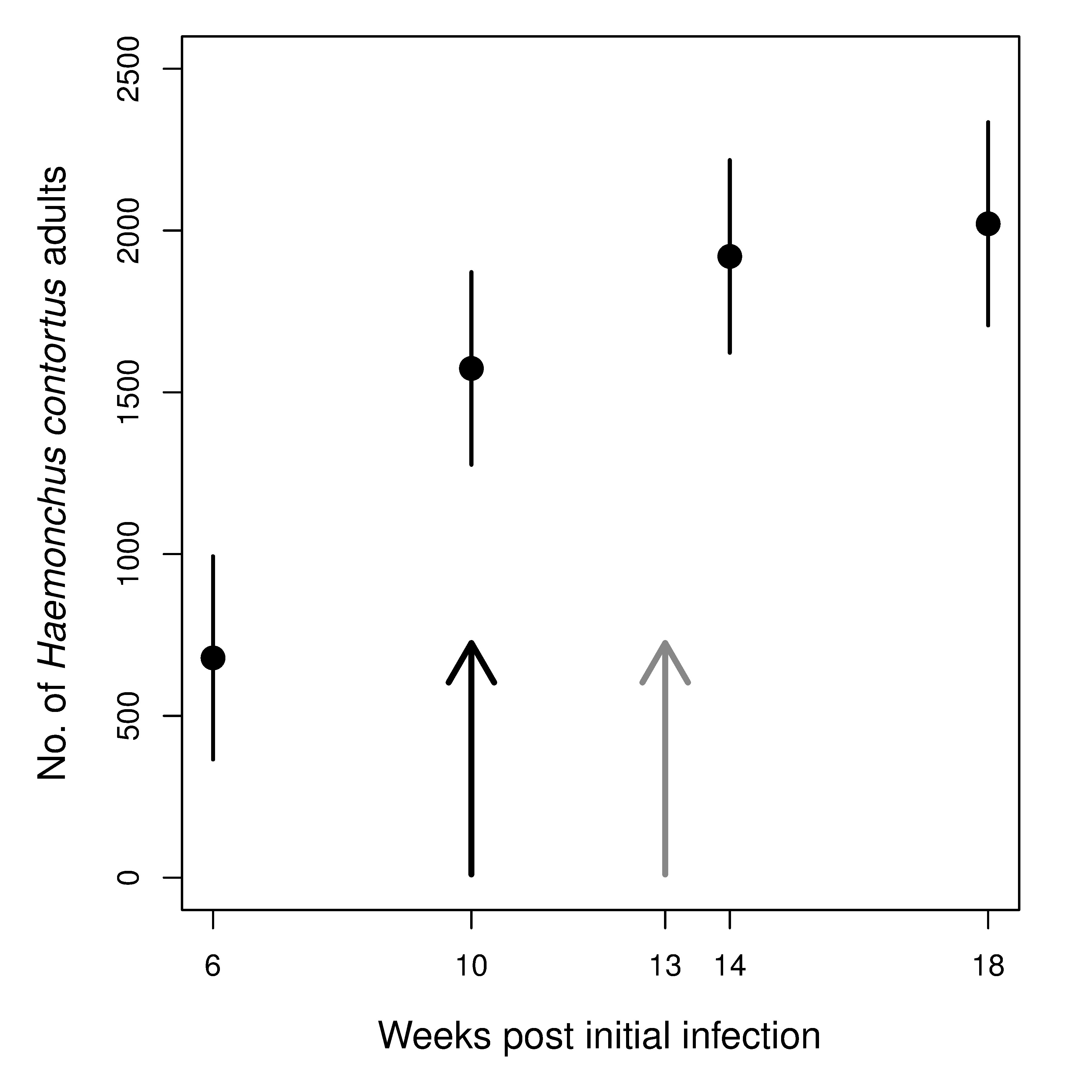

Supplement: S5 Figure [file rspb20172610supp5.tif]

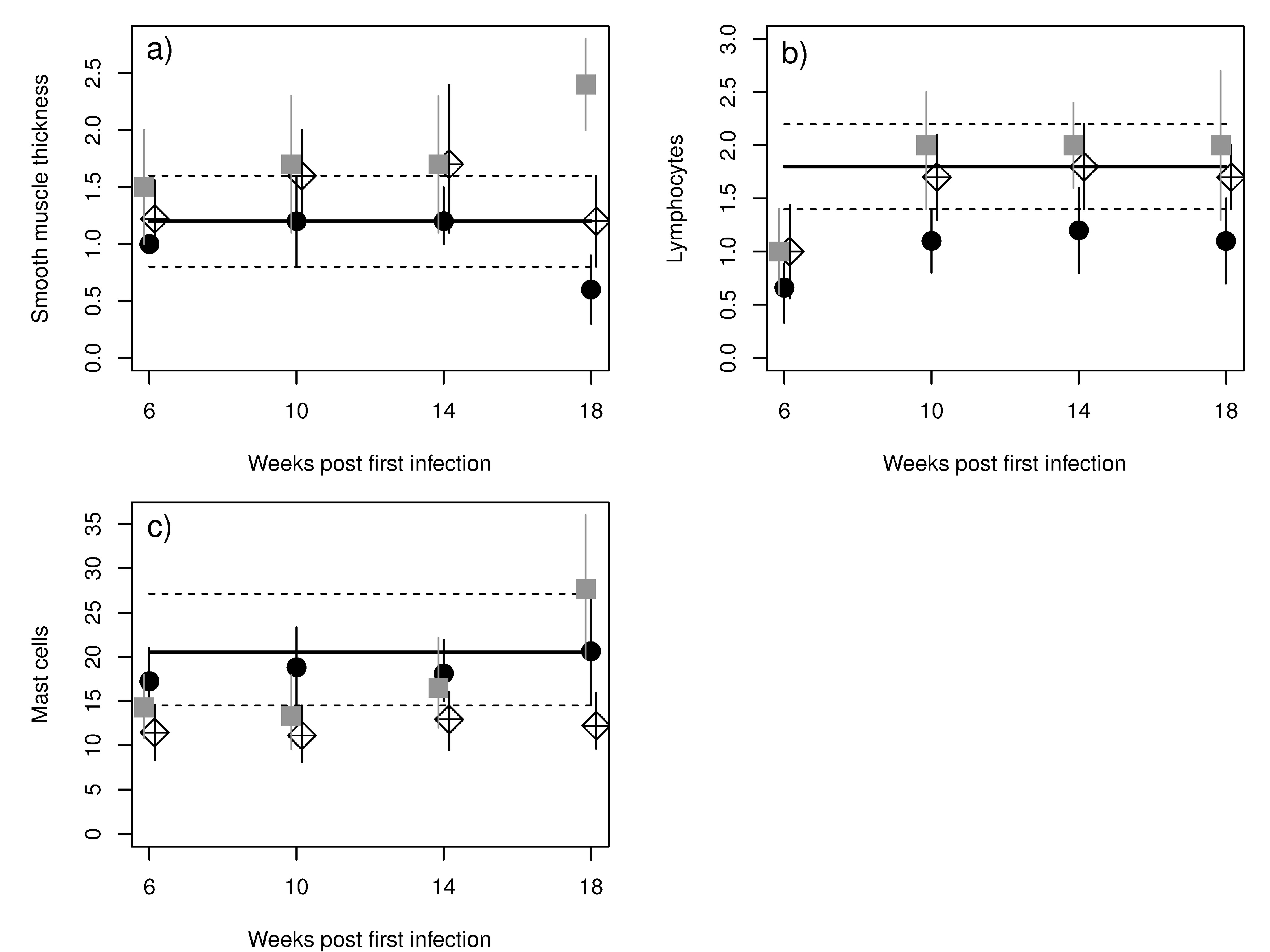

Supplement: S7 Figure [file rspb20172610supp7.tif]

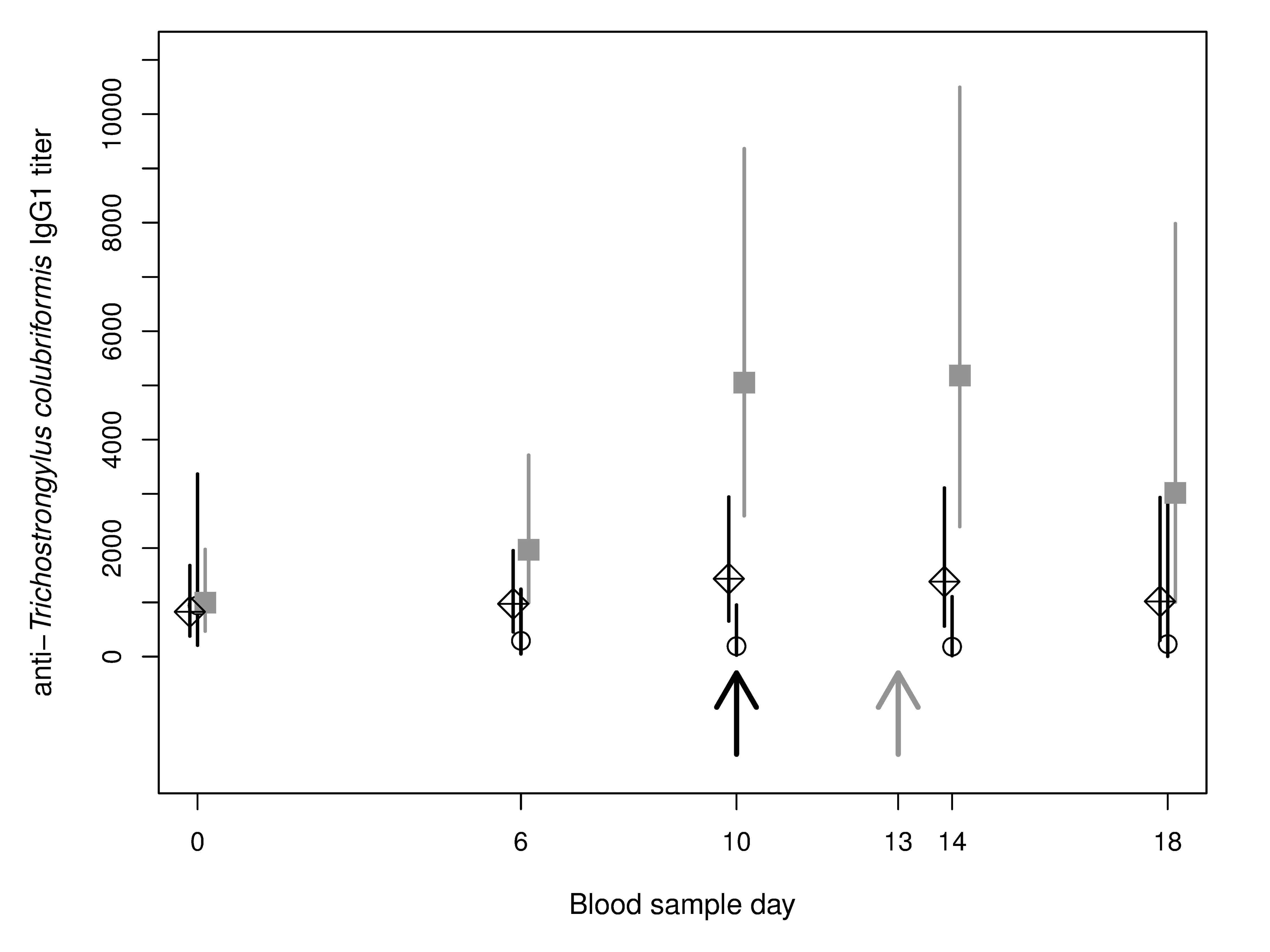

Supplement: S9 Figure [file rspb20172610supp9.tif]
