# Supplementary material for: Predicting the effects of parasite co-infection across species boundaries
Source: Proc Biol Sci. 2018 Mar 14;285(1874):20172610. doi: 10.1098/rspb.2017.2610 (PMC5879626; doi:10.1098/rspb.2017.2610)
Supplement: S6 Table [file rspb20172610supp6.docx]

**S6. Summary information of Principal Component Analysis for the jejunal immune response**. a) Proportion of variance assigned to each principal component axis. b) PCA loading coefficients of jejunal immune components on each principal component axis.

**a.**

|  | **PC1** | **PC2** | **PC3** | **PC4** | **PC5** | **PC6** | **PC7** |
| --- | --- | --- | --- | --- | --- | --- | --- |
| Standard Deviation | 1.849 | 0.929 | 0.907 | 0.840 | 0.659 | 0.636 | 0.591 |
| Proportion of Variance | 0.489 | 0.123 | 0.117 | 0.101 | 0.062 | 0.058 | 0.050 |
| Cumulative Proportion of Variance | 0.489 | 0.612 | 0.729 | 0.830 | 0.892 | 0.950 | 1.000 |

**b.**

| **Immune component** | **PC1** | **PC2** | **PC3** | **PC4** | **PC5** | **PC6** | **PC7** |
| --- | --- | --- | --- | --- | --- | --- | --- |
| Eosinophils | 0.394 | -0.126 | 0.390 | -0.458 | -0.309 | -0.469 | 0.390 |
| Globule leucocytes | 0.391 | 0.118 | -0.166 | 0.627 | -0.244 | -0.525 | -0.278 |
| Goblet cells | 0.397 | 0.396 | 0.254 | -0.326 | 0.456 | -0.004 | -0.554 |
| Goblet cells with granulocytes | 0.333 | 0.487 | -0.558 | -0.254 | -0.382 | 0.322 | 0.164 |
| Lymphocytes | 0.302 | -0.742 | -0.369 | -0.248 | -0.054 | 0.106 | -0.382 |
| Mast cells | 0.382 | -0.112 | 0.516 | 0.343 | -0.261 | 0.624 | 0.009 |
| Smooth muscle thickness | 0.431 | -0.114 | -0.207 | 0.215 | 0.648 | 0.032 | 0.539 |
