# Supplementary material for: Predicting the effects of parasite co-infection across species boundaries
Source: Proc Biol Sci. 2018 Mar 14;285(1874):20172610. doi: 10.1098/rspb.2017.2610 (PMC5879626; doi:10.1098/rspb.2017.2610)
Supplement: S8 Table [file rspb20172610supp8.docx]

**S8. Summary information of Principal Component Analysis for the abomasal immune response.** a) Proportion of variance assigned to each principal component axis. b) PCA loading coefficients of abomasal immune components on each principal component axis.

**a.**

|  | **PC1** | **PC2** | **PC3** | **PC4** |
| --- | --- | --- | --- | --- |
| Standard Deviation | 1.572 | 0.939 | 0.656 | 0.466 |
| Proportion of Variance | 0.618 | 0.221 | 0.107 | 0.054 |
| Cumulative Proportion of Variance | 0.618 | 0.839 | 0.946 | 1.000 |

**b.**

| **Immune component** | **PC1** | **PC2** | **PC3** | **PC4** |
| --- | --- | --- | --- | --- |
| globule leukocytes | 0.455 | 0.586 | -0.641 | 0.197 |
| mast cells | 0.492 | 0.415 | 0.759 | 0.097 |
| eosinophils | 0.480 | -0.629 | -0.046 | 0.610 |
| lymphocytes | 0.566 | -0.299 | -0.106 | -0.761 |
